# Blunted rest-activity circadian rhythm increases the risk of all-cause, cardiovascular disease and cancer mortality in US adults

**DOI:** 10.1038/s41598-022-24894-z

**Published:** 2022-11-30

**Authors:** Yanyan Xu, Shaoyong Su, Xinyue Li, Asifhusen Mansuri, William V. McCall, Xiaoling Wang

**Affiliations:** 1 Georgia Prevention Institute, Medical College of Georgia, Augusta University, Building HS-1715, Augusta, GA 30912 USA; 2 Center for Biotechnology and Genomic Medicine, Medical College of Georgia, Augusta University, Augusta, GA USA; 3 School of Data Science, City University of Hong Kong, Hong Kong, China; 4 Division of Pediatric Nephrology and Hypertension, Children’s Hospital of Georgia, Medical College of Georgia, Augusta University, Augusta, GA USA; 5 Department of Psychiatry and Health Behavior, Medical College of Georgia, Augusta University, Augusta, GA USA

**Keywords:** Public health, Epidemiology, Lifestyle modification, Preventive medicine

## Abstract

To examine whether rest-activity circadian rhythm parameters can predict all-cause, cardiovascular disease and cancer mortality in a general adult population of the US. We further compared the mortality predictive performance of these parameters with that of traditional risk factors. This study included 7,252 adults from US National Health and Nutrition Examination Surveys (NHANES) 2011–2014, who had wrist accelerometer data obtained at baseline and follow-up status linked to the National Death Index records (2011–2019). During a median of 81 months (interquartile range, 69–94 months) of follow-up, 674 (9.3%) deaths occurred. There were inverse associations between relative amplitude (RA) and all-cause mortality, cardiovascular disease and cancer mortality with increased quartiles RA associated with lower mortality risk (all *P* < 0.05). The Hazard Ratios ranged from 0.61 to 0.79. Furthermore, RA outperformed all the tested traditional predictors of all-cause mortality with the exception of age. This study suggests that participants with blunted rest-activity circadian rhythms had a higher risk of all-cause, cardiovascular disease and cancer mortality. Future studies will be needed to test whether interventions that regulate rest-activity circadian activity rhythms will improve health outcomes.

## Introduction

It is well known that circadian rhythm functions to ensure optimal organismal adaptation and plays vital roles in maintaining physiological and homeostatic status and reducing vulnerability to diseases^1,2^. In mammals, the internal circadian rhythm pacemaker is located within hypothalamus, and is entrained to the 24-h light/dark cycle through cues such as light signals. Due to the widespread availability of electrical lighting, we can easily extend the daily activities late into the night. This behavior can shift the endogenous circadian rhythm and thus result in a misalignment between external and endogenous circadian physiology. It is believed that circadian misalignment or disruption^3^ is especially pronounced in night-shift workers and is considered to be linked with different health problems including hypertension, sleep disturbance, diabetes and cardiovascular disease^4^. Circadian disruption can also happen through individual’s choice of daily schedules in a “real life” setting, such as the timing of physical activity, sleep, food intake, etc.^5,6^.

Rest-activity rhythm is one of the most prominent outputs of the circadian system and abnormalities in rest-activity rhythm are now considered as manifestations of long-term mild disruption of circadian rhythm in the daily real-world setting^7,8^. Several measures calculated from rest-activity rhythm are thought to reflect the strength and timing of the circadian systems in real life and these measurements have been associated with multiple human disorders such as increased inflammatory status^6,9^, impaired glucose tolerance^10^, and increased mortality in elderly populations^11,12^ or patients with diseases such as metastatic cancer^13^. Relative amplitude, a parameter indicating the overall robustness of the rest-activity rhythm, has also been reported as the only predictor that outperforms all other traditional predictors of all-cause mortality in the UK Biobank study with the exception of age^14^. However, the association of rest-activity rhythm with cardiovascular disease (CVD) or cancer specific mortality as well as the association between rest-activity rhythm and all-cause mortality in a general adult population of the United States (US) has not been explored. Furthermore, it remains unknown whether the association of disrupted rest-activity rhythm with mortality might actually represent the confounding effect of sleep on mortality, as sleep/wake cycles are intertwined with the circadian system^15^.

We hypothesized that disrupted rest-activity rhythm was associated with an increased risk of all-cause, CVD and cancer mortality and tested this hypothesis in a nationally representative sample of the US population. We also adjusted the sleep parameters objectively obtained from the wrist-worn accelerometers in the analysis, and compared the parameters of rest-activity rhythm with traditional risk factors in terms of their predictive performance for all-cause mortality.

## Methods

This observational study was conducted and reported following recommendation of the Strengthening the Reporting of Observational Studies in Epidemiology (STROBE) statement^16^.

### Sample

**Figure 1:**
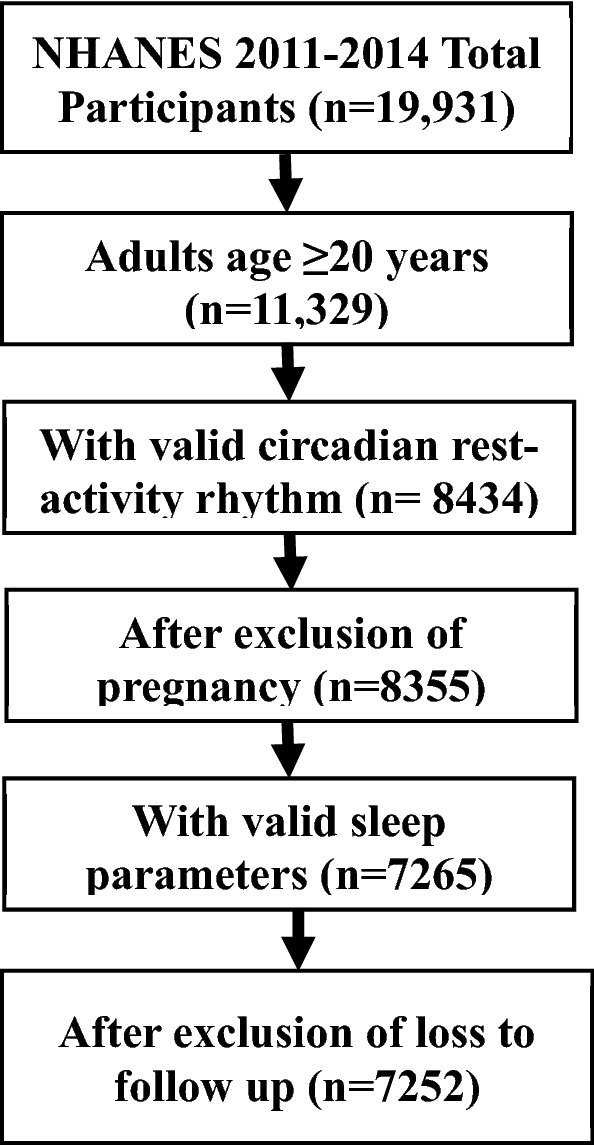
Flowchart for inclusion of study participants.

National Health and Nutrition Examination Survey (NHANES) is an ongoing nationally—representative, cross-sectional survey study conducted by the US Centers for Disease Control and Prevention^17^. NHANES used a multistage probability sampling design to produce a weighted, representative sample of the US population. Wrist accelerometers were incorporated in the 2011–2014 NHANES study cycle, and this is the first time that 24 h accelerometer data are available on a nationally representative sample of US residents. All-cause and cause-specific mortality were assessed in all participants linked to the National Death Index (NDI) mortality data (2011–2019) [dataset]^18^. The National Center for Health Statistics Research Ethics Review Board approved all NHANES protocols, and all participants gave informed consent. This study has been performed in accordance with the Declaration of Helsinki. Figure 1 illustrates the flow of participants selected for inclusion in this analysis. As shown in Supplementary Table 1, the participants included in this study were older, more likely to be female and Non-Hispanic (NH) White and more likely to have a better social economic status as indexed by the ratio of family income to poverty in comparison with the participants that were excluded from this analysis. The majority of the exclusion was caused by invalid rest-activity rhythm data (n = 2895) or the invalid sleep data (n = 1090). Since both of these two datasets were obtained from accelerometer recording, indicating older, female, NH White and participants with a better social economic status have a better compliance to the accelerometer protocol.

### Rest-activity circadian rhythm parameters

Accelerometer recording and data preprocessing were documented previously^6,10^. R package “nparACT” was used to compute the following nonparametric variables of rest-activity rhythms from the summary activity count data, which have been extensively described before^19,20^: (1) Interdaily stability (IS), which estimates how closely the 24-h rest–activity pattern follows the 24-h light–dark cycle (IS ≃ 0 for Gaussian noise, IS ≃ 1 for perfect stability); (2) Intradaily variability (IV), which quantifies the fragmentation of the 24-h rhythm (IV ≃ 0 for a perfect sine wave, IV ≃ 2 for Gaussian noise); (3) The relative amplitude (RA), which is the relative difference between the most active continuous 10-h period (M10) and the least active continuous 5-h period (L5) in an average 24 h (midnight to midnight). It is a nonparametric measure of the amplitude of rest-activity rhythm with higher RAs indicating more robust 24-h rest–activity oscillations, reflecting both higher activity when awake and relatively lower activity during the night; (4) Onset time of the M10 (M10 start time), which indicates the starting time of the peak activity (i.e. the most active period); and (5) Onset time of the L5 (L5 start time), which provides an indication of the starting time of nadir activity (i.e. the less active period). A detailed description on the definition of these parameters were provided in the supplementary document 1.

### Sleep parameters

Sleep parameters were derived from accelerometer summary count data using an unsupervised sleep–wake identification algorithm based on Hidden Markov Model (HMM) as described previously^21,22^. Briefly, the block of the longest sleep period in the day (noon-noon) was identified as the sleep period time (SPT) window. The start of SPT window was defined as the sleep onset time. Wake/activity bouts were identified during the SPT window. Sleep duration was defined using the following equation: sleep duration = the SPT window duration—the summed duration of all wake bouts. Sleep efficiency was calculated as sleep duration divided by the SPT window duration. R code for implementing the HMM algorithm is at https://github.com/xinyue-L/hmmacc. Records with a SPT window duration < 3 h or > 15 h were excluded before the calculation of average sleep parameters for each individual. Individuals with valid sleep parameters less than 3 days were excluded from the analysis.

### Other covariates

Self-reported information about demographic factors regarding age, sex, race (i.e., Non-Hispanic (NH) White, NH Black, Mexican American and other race—including other Hispanic, Asian and other race), smoking status, and family income-to-poverty ratio were collected. Smokers were defined when individuals reported a consumption of ≥ 100 cigarettes during their lifetime. Body mass index (BMI) was calculated as weight in kilograms divided by height in meters squared. Participants were categorized into ideal, intermediate, or poor leisure-time physical activity levels based on whether they met the American Heart Association recommendations for weekly activity based on self-reported physical activity collected by questionnaire^23^: ideal, 75 min or more of vigorous activity or 150 min or more of moderate activity or 150 min or more of combined moderate and vigorous physical activity; intermediate, more than 0 min of physical activity but less than recommendations; and poor, 0 min of physical activity. Self-reported presence of chronic disorders including history of CVD (i.e. congestive heart failure, coronary heart disease, angina pectoris and heart attack), stroke and cancer were also included as study covariates. Educational level was categorized as “ < high school” (including less than 9th grade and 9–11th grade, which includes 12th grade with no diploma), “high school” (including high school grad/GED or equivalent) and “college and above” (including some college or AA degree and college graduate or above). Alcohol drinking was defined if participants had at least 12 alcohol drinks/1 year. Self-reported general health information was used to categorize the participants to a “good” health status if they reported an “excellent/very good/good” condition, with “fair/poor” defined as the other group.

### Statistical analysis

STATA (v16) was used to perform survey data analysis to account for complex survey design and produce representative estimates of the US population. Four-year survey weights were calculated and used in all analyses to adjust for unequal selection probability and non-response bias in accordance with NHANES analytical guidelines. Descriptive statistics were presented as population means, and standard deviations for continuous variables and weighted proportions for categorical variables. The variables were listed according to the ranking of their predictive performance of all-cause mortality based on the Concordance estimated from univariate Cox regression models^24^. Concordance is a weighted average of time-dependent incident/dynamic area under the receiver operating characteristic curve. Concordance ranges from 0 to 1 indicating a perfectly discordant to a perfectly concordant risk score, and a value of 0.5 indicating the risk score is independent of the event times^25^. Hazard Ratios (HRs) and 95% confidence intervals (CI) were estimated for all-cause mortality, CVD and cancer-specific death risk for each rest-activity circadian rhythm parameters using time (months) from NHANES Mobile Examination Center (MEC) date to mortality or censoring. Separate models were fitted for all-cause mortality and each cause-specific mortality, and competing risks were taken into account. We tested 3 models for each rest-activity rhythm parameters with increased number of covariates. Baseline model (model 1) included age, sex, and race as covariates. Model 2 further adjusted ratio of family income to poverty level, smoking status, physical activity, education level, alcohol consumption, sleep efficiency, and sleep duration. Model 3 further included general health, BMI, history of hypertension, CVD, cancer, diabetes and stroke as covariates. Covariates were selected for multivariable models based on known or suspected confounders for the association between rest-activity circadian rhythm and mortality. Non-linear effects, or time-varying effects were not considered. To compare the parameters of rest-activity rhythm with traditional risk factors in terms of their predictive performance for all-cause mortality, we selected the best set of predictors using forward selection. Variables are included sequentially based on the net change in the tenfold cross-validated concordance^24–26^. Briefly, the data were randomly divided into 10 sets, with the model fitting conducted in 90% of the sample and the rest 10% of the sample for validation. The average results across 10 rounds were used to represent the model’s overall performance. Because a one-unit change in RA and IS or a two-unit change in IV would reflect the difference between the extreme lower and upper ends of the range, they were divided into quartiles for the regression models. A 2-sided *P* < 0.05 was considered statistically significant. The interactions between sex/race and rest-activity rhythm parameters were also tested to examine whether the associations of rest-activity circadian rhythm parameters with mortality risk were modified by sex/race.

### Ethics approval and consent to participate

The NHANES protocols were approved by the National Center for Health Statistics Ethics Review Board (Protocol# 2011–17) and all participants provided written informed consent.

## Results

Our analytical sample included 7252 participants aged ≥ 20 years (median [IQR]: 49 [36–62]), which is representative of 153.9 million noninstitutionalized residents of the United States. In this study, during a median of 81 months (interquartile range, 69–94 months) of follow-up, 674 deaths occurred, including 213 from CVD and 156 from cancer. Baseline characteristics of the 7252 participants by mortality status are displayed in Table 1. Variables were ranked in the decreasing order of their predictive performance. Age is the strongest predictor of mortality with hypertension, IV, RA, physical activity and sleep efficiency being the next second to fifth predictors respectively.

**Table 1:** Population characteristics stratified by mortality status at follow-up ordered by estimated concordance from univariate cox regressions (n = 7252).

Rank	Characteristics	Alive (N = 6578)	Deceased (N = 674)	Concordance
1	Age, mean (SD)	47.8 (16.0)	68.4 (13.6)	**0.823**
2	Hypertension, N (%)	2361 (33.2)	467 (66.5)	**0.659**
3	IV, mean (SD)	0.69 (0.2)	0.83 (0.27)	**0.642**
4	RA, mean (SD)	0.86 (0.1)	0.81 (0.1)	**0.628**
5	Physical activity, N (%)			**0.621**
	Poor	3231 (45.1)	495 (72.6)	
	Intermediate	1172 (18.8)	65 (9.4)	
	Ideal	2175 (36.1)	114 (18.0)	
6	Sleep efficiency, mean (SD)	0.95 (0.04)	0.94 (0.02)	**0.619**
7	CVD, N (%)	437 (6.0)	199 (28.6)	**0.612**
8	Race, N (%)			**0.612**
	NH White	2601 (66.8)	400 (79.2)	
	NH Black	1479 (10.6)	146 (9.9)	
	Mexican American	835 (8.9)	49 (4.1)	
	Other	1663 (13.8)	79 (6.8)	
9	General health (good), N (%)	4815 (83.8)	382 (62.9)	**0.594**
10	M10 start time, mean (SD)	09:11 (133 min)	08:28 (120 min)	**0.587**
11	Sleep onset, mean (SD)	22:54 (96 min)	22:30 (102 min)	**0.586**
12	Diabetes, N (%)	786 (9.2)	205 (28)	**0.585**
13	Cancer, N (%)	554 (10.1)	164 (25.8)	**0.579**
14	Smoker, N (%)	2743 (43)	391 (59)	**0.578**
15	Education level, N (%)			**0.572**
	< High school	1381 (15.0)	213 (23.1)	
	High schoo/equivalent	1461 (21.3)	166 (25.9)	
	≥ College	3734 (63.7)	292 (51.1)	
16	Stroke, N (%)	174 (2.1)	105 (13.3)	**0.563**
17	Ratio of family income to poverty, mean (SD)	3.0 (1.7)	2.5 (1.5)	**0.560**
18	L5 start time, mean (SD)	00:44 (90 min)	00:31 (84 min)	**0.549**
19	Sleep duration, N (%)			**0.549**
	6 h ≤ Sleep time ≤ 9 h	5126 (77.9)	457(69)	
	Sleep time < 6 h	233 (3.1)	28 (4.4)	
	Sleep time > 9 h	1219 (19.0)	189 (26.6)	
20	Female, N (%)	3511 (53.6)	315 (50.0)	0.530
21	Alcohol drinking, N (%)	4444 (72.3)	442 (72.3)	0.517
22	IS, mean (SD)	0.61 (0.12)	0.60 (0.12)	0.502
23	BMI, mean (SD)	29.2 (6.8)	29.3 (7.6)	0.473

*IQR* Interquartile range; *NH* Non-Hispanic; *BMI* Body mass index; *RA* Relative amplitude; *IS* Interdaily stability; *IV* Intradaily variability; *M10* The most active continuous 10-h period; *L5* The least active continuous 5-h period.

% and means (SD)/medians (IQR) were weight adjusted.

Bold denoted a statistical significance in the univariate cox regressions.

The associations between rest-activity circadian rhythm parameters with all-cause and cause specific mortality were presented in Table 2. Across all three models, increased levels of RA were associated with lower risk of all-cause mortality, CVD mortality, and cancer mortality (all *P* < 0.05) with a HR ranging from 0.61 to 0.79. In the baseline model adjusting for age, race and sex, increased levels of IV (increased fragmentation) were associated with increased all-cause mortality (HR = 1.22; 95% CI: 1.11–1.34) and CVD-cause mortality (HR = 1.3; 95% CI: 1.1–1.57). Further adjustment for socio-demographic, behavior and health factors attenuated the associations, but the significant associations remained for model 2 (HR = 1.18; 95% CI: 1.07–1.31) and model 3 (HR = 1.13; 95% CI: 1.01–1.26) for all-cause mortality. We did not observe consistently significant associations of IS, M10 start time or L5 start time with mortality. We did not observe interactions between sex/race and rest-activity rhythm on the prediction of mortality.

**Table 2:** Associations of rest-activity parameters with all-cause and cause-specific mortality.

Causes of mortality	Rest-activity rhythm parameters	Model 1		Model 2		Model 3	
		HR (95% CI)	*P*	HR (95% CI)	*P*	HR (95% CI)	*P*
All-cause	RA	**0.63 (0.55, 0.72)**	** < 0.0001**	**0.70 (0.60, 0.82)**	** < 0.0001**	**0.75 (0.63, 0.89)**	**0.002**
	IV	**1.22 (1.11, 1.34)**	** < 0.0001**	**1.18 (1.07, 1.31)**	**0.002**	**1.13 (1.01, 1.26)**	**0.041**
	IS	**0.84 (0.75, 0.94)**	**0.003**	0.94 (0.83, 1.06)	0.276	0.934 (0.823, 1.06)	0.277
	M10 start time	1.03 (0.98, 1.08)	0.245	1.01 (0.96, 1.06)	0.628	1.01 (0.96, 1.06)	0.833
	L5 start time	1.03 (0.96, 1.1)	0.42	0.97 (0.90, 1.04)	0.375	0.967 (0.90, 1.04)	0.340
CVD-cause*	RA	**0.61 (0.50, 0.74)**	** < 0.0001**	**0.73 (0.58, 0.91)**	**0.005**	**0.77 (0.61, 0.99)**	**0.042**
	IV	**1.3 (1.1, 1.57)**	**0.014**	1.20 (0.95, 1.51)	0.127	114 (0.90, 1.45)	0.289
	IS	**0.79 (0.70, 0.89)**	** < 0.0001**	**0.87 (0.77, 0.98)**	**0.030**	0.89 (0.76, 1.03)	0.128
	M10 start time	0.97 (0.91, 1.02)	0.231	**0.94 (0.89, 0.99)**	**0.031**	**0.93 (0.86, 0.99)**	**0.023**
	L5 start time	0.97 (0.89, 1.07)	0.57	0.94 (0.86, 1.02)	0.123	0.92 (0.83, 1.02)	0.127
Cancer-cause*	RA	**0.79 (0.64, 0.97)**	**0.022**	**0.69 (0.52, 0.92)**	**0.013**	**0.70 (0.51, 0.95)**	**0.024**
	IV	0.93 (0.79, 1.1)	0.358	0.93 (0.79, 1.1)	0.370	0.92 (0.78, 1.1)	0.359
	IS	1.04 (0.84, 1.30)	0.703	1.07 (0.83, 1.38)	0.612	1.06 (0.82, 1.36)	0.662
	M10 start time	1.03 (0.89, 1.19)	0.674	1.02 (0.87, 1.2)	0.814	1.02 (0.87, 1.2)	0.797
	L5 start time	1.01(0.86, 1.18)	0.95	0.97 (0.82, 1.14)	0.702	0.97 (0.82, 1.14)	0.705

Model 1: Adjusted for age at baseline, race, sex.

Model 2: Adjusted for age at baseline, race, sex, ratio of family income to poverty, education, physical activity, smoking, alcohol drinking, sleep efficiency, sleep duration.

Model 3: Adjusted for age at baseline, race, sex, ratio of family income to poverty, education, physical activity, smoking, alcohol drinking, sleep efficiency, sleep duration, general health, BMI, hypertension, CVD, cancer, stroke, diabetes.

*RA* Relative amplitude; *IS* Interdaily stability; *IV* Intradaily variability; *M10* The most active continuous 10-h period; *L5* The least active continuous 5-h period.

*Model 3: Adjusted for age at baseline, race, sex, ratio of family income to poverty, education, physical activity, smoking, alcohol drinking, sleep efficiency, sleep duration, general health, BMI, hypertension.

Significant values are in [bold].

The results of prediction performance for all-cause mortality were presented in Table 3. The forward selection at each step of the procedure was performed using candidates including all the traditional risk factors and the rest-activity circadian rhythm parameters as well as the sleep parameters from model 3 mentioned above. Variables were accepted into the model one at a time according to which increased concordance the most. Two stopping rules were used: when the concordance did not increase by more than 0.005 or by more than 0.001. Variables were ordered according to their inclusion order. Coefficient estimates and 95% confidence intervals were shown for each model associated with a stopping rule, respectively. For the more conservative stopping rule (δ C ≥ 0.005), only 3 variables were selected, which were age, RA, and self-reported general health. For the less conservative stopping rule (δ C ≥ 0.001), four additional factors were included, which were CVD, physical activity, the ratio of family income to poverty, and race. No sleep parameters or other rest-activity circadian rhythm parameters were selected using this procedure.

**Table 3:** Results of forward selection using a set of predictor variables based on improvement in concordance (δC).

Variable	Cumulative concordance	δC	
**Stopping rule: δC ≥ 0.005**			
Age	0.8225		0.089 (0.079, 0.098)
RA	0.8315	0.0090	− 0.377 (− 0.507, − 0.248)
General health	0.8374	0.0059	
(Very) good/excellent			− 0.799 (− 0.985, − 0.614)
Fair/poor			Ref.
**Stopping rule: δC ≥ 0.001**			
Age	0.8225		0.082 (0.074, 0.092)
RA	0.8315	0.0090	− 0.333 (− 0.481, − 0.185)
General health	0.8374	0.0059	
(Very) good/excellent			− 0.590 (− 0.814, − 0.367)
Fair/poor			Ref.
CVD	0.8408	0.0034	0.325 (0.044, 0.606)
Physical activity	0.8431	0.0023	
Poor			Ref.
Intermediate			− 0.577 (− 0.917, − 0.237)
Ideal			− 0.425 (− 0.778, − 0.073)
Ratio of family income to poverty	0.8451	0.0020	− 0.114 (− 0.214, − 0.013)
Race	0.8480	0.0028	
NH White			Ref.
NH Black			− 0.237 (− 0.619, 0.145)
Mexican American			− 0.411 ( − 0.789, − 0.025)
Other			− 0.520 (− 0.878, − 0.162)

*RA* Relative amplitude; *NH* Non-Hispanic. Variables were listed in order of their inclusion into the model and the tenfold cross-validated concordance from a model which included all variables up to the current variable that was presented in the second column. The fourth column presented the estimated coefficient and 95% CIs obtained from the final models fit for each stopping rule. RA has been divided into quartiles.

## Discussion & conclusions

In the present study, for the first time in a nationally representative adult sample, we observed that blunted rest-activity rhythm, indexed by decreased levels of relative amplitude, was associated with all-cause mortality as well as CVD—and cancer-specific mortality. Those associations were independent of age, sociodemographic factors, health status and objective measurements of sleep behaviors. Furthermore, after excluding participants with diabetes, CVD, cancer or stroke at the baseline visit, our finding stays the same for the all-cause mortality (Supplementary Table 2), indicating that impaired robustness of rest-activity circadian rhythm was associated with increased risk of all-cause mortality even in the relatively healthy population. We further compared the prediction performance of the rest-activity circadian rhythm with sleep parameters and other traditional risk factors, and found relative amplitude to be predictive of mortality above and beyond traditional predictors with the exception of age. Our results indicate overall robustness of circadian rhythm is a strong predictor of mortality in the general population.

Our results are in consistent with the findings from the UK Biobank study^14^ and the Rotterdam Study^27^ for the association of rest-activity rhythm with all-cause mortality as well as the findings from the Study of Osteoporotic Fractures (SOF) in elderly women (mean age 84 years)^12^ and the MrOS Sleep Study in elderly men (mean age 76 years)^11^ for the association of rest-activity rhythm with all-cause mortality and CVD mortality. In the SOF study, adding total sleep time or sleep efficiency to the models did not change the associations between rest-activity rhythm parameters and mortality. Zuurbier et al.^27^ specifically compared the prediction performance of rest-activity circadian rhythm parameters with both subjectively and objectively measured sleep parameters in the Rotterdam Study and observed that sleep measures were not related to mortality after adjustment for health parameters. In line with their results, we also observed that relative amplitude, the index of the overall robustness of the rest-activity circadian rhythm, was independently and more strongly associated with mortality than sleep parameters. In consideration of the relatively strong correlation between relative amplitude and sleep efficiency (r = 0.53, *P* < 0.001, Supplementary Table 3, we redid model 2 and model 3 without including sleep efficiency as a covariate to avoid the potential issue of over adjustment and listed the results in Supplementary Table 4. The results remained the same. This suggests that although sleep/wake cycles are intertwined with the circadian system, the circadian rhythm has its independent role in the risk of mortality.

The mechanism underlying the association of rest-activity circadian rhythms with mortality cannot be interpreted based on these observational data. However, animal and human laboratory studies have shown that the number of neurons in the suprachiasmatic nucleus (SCN), where the endogenous clock locates, decreased along with age^28–30^. This may lead to a decreased amplitude of circadian rhythm, and a decreased ability of the SCN to drive synchronization, causing a complex deviation from homeostasis, and driving risks for mortality with the aging process. In addition to the normal aging process, disrupted rest-activity circadian rhythm has also been suggested to play a role in multiple disorders such as diabetes^31^, depression^32^, dementia^33–35^ and cancer^36^ as well as pro-inflammatory status^6,9^ and pro-diabetes^10^, indicating disrupted rest-activity rhythm may also serve as a biomarker for accelerating biological aging. On the other hand, rest-activity rhythm parameters also reflect a combination of the interaction between the endogenous circadian rhythm and rhythmic environmental and behavioral factors. It has been suggested that the rest-activity circadian rhythm could be modified through environmental and lifestyle changes^37,38^. Future studies are warranted to examine whether environmental and lifestyle interventions or pharmacological treatments may correct the disrupted circadian rhythm and thus reduce the risk of mortality.

Our study has several strengths. NHANES population is from a nationally representative sample, which increases the generalizability of our findings. The linked mortality data is through death certificate record from the National Death Index, with minimal missing on the link. The large number of events for the outcomes studied and well-characterized data on multiple risk factors and confounders, provide us the opportunity to comprehensively compare the prediction effect of rest activity rhythm parameters with the traditional risk factors on mortality risk. Nevertheless, there are limitations. First of all, changes in rest-activity circadian parameters over time were not accounted for in the analyses, which might have underestimated the magnitude of the associations (regression dilution bias). Second, there may be other residual confounding factors that were not included, although we have adjusted for multiple confounding factors at baseline. Third, we did not observe consistent associations between rest-activity circadian parameters and other cause specific (except CVD and cancer) mortality, and this may due to the limited sample size of the cause-specific death events. Furthermore, we did not perform adjustment for multiple testing (i.e. a *P* < 0.05 was defined as statistically significance) in that the current study is a traditional, observational epidemiology study. Although the debate of whether *P* values should be corrected for multiple comparisons has been a long one, the dominant view to-date for most applications of traditional epidemiology is that there is no need to adjust for multiple comparisons in observational epidemiology. However, with the increased numbers of exposures (i.e. exposomes) being tested and the lack of public preregistration of hypotheses, being transparent about the extent of multiplicity is becoming important for the understanding of the results^39^. For the current study which includes 5 rest-activity rhythm parameters, a Bonferroni correction will result in a *P* value threshold of 0.01. With this threshold, the associations of rest-activity rhythm with CVD and cancer mortality become inconsistent across models and this piece of finding needs to be further replicated.

In summary, we observed in a nationally representative adult sample, participants with disrupted rest-activity circadian rhythms had higher risk of all-cause mortality. Future studies will be needed to confirm the link between rest-activity rhythms and CVD or cancer specific mortality and to test whether interventions (e.g. the arrangements of physical activity during the day/night, the sleep/wake cycle, and bright light exposure) that regulate rest-activity circadian activity rhythms will improve health outcomes.

## Supplementary Information

Supplementary Information 1.

Supplementary Information 2.

## Data Availability

The NHANES data that support the findings of this study are available from CDC centers for disease control and prevention website [hyperlink https://wwwn.cdc.gov/nchs/nhanes/Default.aspx].

## Abbreviations

CVD: Cardiovascular disease
NH: Non-Hispanic
CI: Confidence interval
NHANES: The US National health and nutrition examination survey
RA: Relative amplitude
IS: Interdaily stability
IV: Intradaily variability
SD: Standard deviation
NDI: National death index
SPT: Sleep period time
BMI: Body mass index
HR: Hazard ratio
IQR: Interquartile range
