# Supplementary material for: Blunted rest-activity circadian rhythm increases the risk of all-cause, cardiovascular disease and cancer mortality in US adults
Source: Sci Rep. 2022 Nov 30;12:20665. doi: 10.1038/s41598-022-24894-z (PMC9712599; doi:10.1038/s41598-022-24894-z)
Supplement: Supplementary file 1 — Supplementary Information 1. [file 41598_2022_24894_MOESM1_ESM.docx]

Supplementary Table 1. Comparisons in the general characteristics between the participants included and the participants excluded from this study

| Characteristics | Included (N=7252) | Excluded (N=3955) | P-value |
| --- | --- | --- | --- |
| Age (years), median (IQR) | 49 (36-62) | 42 (29-56) | <0.001 |
| Race, N (%) |  |  | <0.001 |
| NH White | 3001 (67.7) | 1471 (63) |  |
| NH Black | 1625 (10.5) | 969 (13.2) |  |
| Mexican American | 884 (8.5) | 413 (8.3) |  |
| Other | 1742 (13.2) | 1102 (15.5) |  |
| Education level, N (%) |  |  | 0.645 |
| <High school | 1594 (15.6) | 962 (17.0) |  |
| High school/equivalent | 1627 (21.6) | 825 (19.8) |  |
| ≥College | 4026 (62.7) | 2161 (63.2) |  |
| Ratio of family income to poverty, median (IQR) | 2.9 (1.35-5) | 2.6 (1.2-4.9) | 0.009 |
| Female, N (%) | 3826 (53.4) | 1883 (47.2) | 0.001 |

Participants who were pregnant at the time of examination were not included in this table.

% and means (SD) /medians (IQRs) were weight adjusted

IQR: interquartile range; NH: Non-Hispanic; BMI, body mass index

Supplementary Table 2. Association of RA with all-cause mortality in participants without diabetes, CVD, cancer and stroke at baseline.

| Causes of mortality | Rest-activity rhythm parameters |  | Model 1 | |  | Model 2 | |  | Model 3 | |
| --- | --- | --- | --- | --- | --- | --- | --- | --- | --- | --- |
|  |  |  | HR (95% CI) | *P* |  | HR (95% CI) | *P* |  | HR (95% CI) | *P* |
| All-cause | RA |  | **0.67 (0.55, 0.81)** | **<0.0001** |  | **0.71 (0.57, 0.90)** | **<0.006** |  | **0.72 (0.57, 0.92)** | **0.01** |

N=5290 for model 1; N=4538 for model 2; N=4516 for model 3

Model 1: Adjusted for age at baseline, race, sex

Model 2: Adjusted for age at baseline, race, sex, ratio of family income to poverty, education, physical activity, smoking, alcohol drinking, sleep efficiency, sleep duration

Model 3: Adjusted for age at baseline, race, sex, ratio of family income to poverty, education, physical activity, smoking, alcohol drinking, sleep efficiency, sleep duration, general health, BMI, hypertension

**Supplementary Table 3. Spearman’s correlations between rest-activity rhythm parameters and sleep parameters**

|  | Sleep Efficiency |  | Sleep Duration |
| --- | --- | --- | --- |
|  | Correlation |  | Correlation |
| RA | 0.53*** |  | 0.036*** |
| IS | 0.17*** |  | 0.046*** |
| IV | -0.086*** |  | -0.099*** |
| M10 start time | -0.016 |  | 0.0192 |
| L5 start time | -0.050*** |  | 0.027* |

*p<0.05, **p<0.01, ***p<0.001

**Supplementary Table4. Associations of rest-activity parameters with all-cause and cause-specific mortality without sleep efficiency as a covariate**

| Causes of mortality | Rest-activity rhythm parameters |  | Model 2 | | |  | | Model 3 | |
| --- | --- | --- | --- | --- | --- | --- | --- | --- | --- |
|  |  |  | HR (95% CI) | | *P* | |  | HR (95% CI) | *P* |
| All-cause | RA |  | **0.68 (0.59, 0.78)** | | **<0.0001** | |  | **0.72 (0.61, 0.84)** | **<0.0001** |
|  | IV |  | **1.21 (1.09, 1.33)** | | **<0.0001** | |  | **1.14 (1.02, 1.28)** | **0.027** |
|  | IS |  | 0.90 (0.80, 1.01) | | 0.071 | |  | 0.90 (0.79, 1.03) | 0.114 |
|  | M10 start time |  | 1.01 (0.97, 1.06) | | 0.569 | |  | 1.01 (0.96, 1.06) | 0.807 |
|  | L5 start time |  | 0.97 (0.90, 1.05) | | 0.476 | |  | 0.97 (0.90, 1.05) | 0.409 |
| CVD-cause* | RA |  | | **0.69 (0.56, 0.85）** | **<0.0001** | |  | **0.73 (0.57, 0.92）** | **0.009** |
|  | IV |  | 1.23 (0.98, 1.55) | | 0.080 | |  | 1.16 (0.92, 1.48) | 0.212 |
|  | IS |  | **0.83 (0.73, 0.95)** | | **0.007** | |  | 0.85 (0.72, 1.0) | 0.049 |
|  | M10 start time |  | **0.94 (0.89, 0.997)** | | **0.040** | |  | **0.92 (0.86, 0.99)** | **0.032** |
|  | L5 start time |  | 0.94 (0.85, 1.03) | | 0.199 | |  | 0.92 (0.82, 1.04) | 0.175 |
| Cancer-cause* | RA |  | **0.78 (0.61, 0.99)** | | **0.044** | |  | 0.77 (0.60, 1.00) | **0.050** |
|  | IV |  | 0.92 (0.78, 1.09) | | 0.351 | |  | 0.92 (0.78, 1.1) | 0.359 |
|  | IS |  | 1.07 (0.84, 1.38) | | 0.579 | |  | 1.06 (0.83, 1.35) | 0.649 |
|  | M10 start time |  | 1.02 (0.87, 1.19） | | 0.820 | |  | 1.03 (0.87, 1.2) | 0.798 |
|  | L5 start time |  | 0.97 (0.82, 1.14) | | 0.689 | |  | 0.97 (0.82, 1.14) | 0.699 |

Model 2: Adjusted for age at baseline, race, sex, ratio of family income to poverty, education, physical activity, smoking, alcohol drinking, sleep duration

Model 3: Adjusted for age at baseline, race, sex, ratio of family income to poverty, education, physical activity, smoking, alcohol drinking, sleep duration, general health, BMI, hypertension, CVD, cancer, stroke, diabetes

*Model 3: Adjusted for age at baseline, race, sex, ratio of family income to poverty, education, physical activity, smoking, alcohol drinking, sleep duration, general health, BMI, hypertension.
