# Supplementary material for: Blunted rest-activity circadian rhythm increases the risk of all-cause, cardiovascular disease and cancer mortality in US adults
Source: Sci Rep. 2022 Nov 30;12:20665. doi: 10.1038/s41598-022-24894-z (PMC9712599; doi:10.1038/s41598-022-24894-z)
Supplement: Supplementary file 2 — Supplementary Information 2. [file 41598_2022_24894_MOESM2_ESM.docx]

**Supplementary document**

**Blunted rest-activity circadian rhythm increases the risk of all-cause, cardiovascular disease and cancer mortality in US adults**

Author affiliations:

Yanyan Xu^ab^, Shaoyong Su^a^, Xinyue Li^c^, Asifhusen Mansuri^d^, William V. McCall^e^, Xiaoling Wang*^ab^

a Georgia Prevention Institute, Medical College of Georgia, Augusta University, Augusta, GA, USA.

b Center for Biotechnology and Genomic Medicine, Medical College of Georgia, Augusta University, Augusta, GA, USA.

c School of Data Science, City University of Hong Kong, Hong Kong, China.

d Division of Pediatric Nephrology and Hypertension, Children’s Hospital of Georgia, Medical College of Georgia, Augusta University, Augusta, GA, USA.

e Department of Psychiatry and Health Behavior, Medical College of Georgia, Augusta University, Augusta, GA, USA.

Corresponding author: Xiaoling Wang ([xwang@augusta.edu](mailto:xwang@augusta.edu))

**The definitions of rest-activity rhythm parameters [1, 2]**

(a) Interdaily stability is described as the ratio between the variance of the average 24-hour pattern around the mean and the overall variance. The IS evaluates individual days to quantify the similarity of activity patterns. Higher values are indicative of rhythm stability.

The formula for interdaily stability:

$$\frac{N\Sigma_{h=1}^{p}\left( \bar{x}_{h}-\bar{x} \right)^{2}}{p\Sigma_{ⅈ=1}^{N}\left( x_{i}-\bar{x} \right)^{2}}$$

N is the total amount of sampling points, *p* is the number of data per day; $\bar{x}_{h}$ is the hourly means, $x_{i}$ is the individual data points, and $\bar{x}$ is the grand average of all data.

(b) Intradaily variability is calculated as the ratio of the mean squares of the difference between successive hours and the mean squares around the grand mean, as shown below

$$\frac{N\Sigma_{ⅈ=2}^{N}\left( x_{i}-x_{i-1} \right)^{2}}{\left( N-1 \right)\Sigma_{ⅈ=1}^{N}\left( x_{i}-\bar{x} \right)^{2}}$$

(c) RA is the relative difference of M10 and L5. M10: 10 hours with maximal activity, L5: five hours with minimal activity. Minute-wise averages are first calculated across days, and averages for five and ten hours are computed (e.g. for L5 from 6:00 to 11:00, from 6:01 to 11:01 and so on) across 24 h yielding a total of 1440 values. From these, the maximal value from the 10 h averages and the minimal value from the five hour averages are picked as well as the times of the day when M10 and L5 start. The formula for RA:

$$\frac{\left( M10-L5 \right)}{\left( M10+L5 \right)}$$

References:

1. Blume C, Santhi N, Schabus M: **'nparACT' package for R: A free software tool for the non-parametric analysis of actigraphy data**. *MethodsX* 2016, **3**:430-435.10.1016/j.mex.2016.05.006

2. Goncalves BS, Adamowicz T, Louzada FM, Moreno CR, Araujo JF: **A fresh look at the use of nonparametric analysis in actimetry**. *Sleep Med Rev* 2015, **20**:84-91.10.1016/j.smrv.2014.06.002
